# Novel *Z*_*eff*_ imaging method for deep internal areas using back-scattered X-rays

**DOI:** 10.1038/s41598-019-54907-3

**Published:** 2019-12-11

**Authors:** Akio Yoneyama, Masahide Kawamoto, Rika Baba

**Affiliations:** 1 SAGA Light Source, 8-7 Yayoigaoka, Tosu, 841-0005 Japan; 2 Research and Development Group, Hitachi Ltd., 1-280 Higashi-koigakubo, Kokubunji, 185-8601 Japan

**Keywords:** Applied physics, X-rays, Imaging techniques

## Abstract

Elemental kinds, composition ratios, effective atomic number (*Z*_*eff*_), and spatial distributions are the most basic information on materials and determine the physical and chemical properties of materials. X-ray fluorescence analysis have conventionally been used for elemental mapping, however maps on deep internal areas cannot be obtained because the escape depth of fluorescence X-rays is limited to a few mm from the surface of samples. Herein, we present a novel *Z*_*eff*_ imaging method that uses back-scattered X-rays. The intensity ratio of elastic and inelastic back-scattered X-rays depends on the atomic number (*Z*) of a single-element sample (*Z*_*eff*_ for a plural-element sample), and so *Z*_*eff*_ maps in deep areas can be obtained by spectrum analysis of the scattered high-energy incident X-rays. We demonstrated the feasibility of observing a phantom covered by an aluminum plate by using synchrotron radiation X-ray. A fine *Z*_*eff*_ map that can be used to identify materials was obtained from only front-side observation. The novel method opens up a new way for *Z*_*eff*_ mapping of deep areas of thick samples from front-side observation.

## Introduction

Physical and chemical properties of materials depend mainly on the elemental kind, composition ratios, effective atomic number (*Z*_*eff*_), and spatial distribution of samples. Therefore, elemental measurement is indispensable, from developing advanced materials and devices, such as magnets, thermoelectric transducers, power switching devices, and batteries, to revealing the biomedical functions of cells and lipid bilayers and the pathogenesis of many diseases. X-ray photoelectron spectroscopy and X-ray fluorescence analysis, which are used to identify elements by analyzing the energy of emitted photoelectrons and fluorescent X-rays, respectively, are conventional non-destructive measurement methods. The energies of photoelectrons and fluorescence X-rays have specified values that depend on the element, and the absorption length (escape depth) of each material is uniquely determined from a few nm to mm from the surface. Therefore, elemental information for internal areas deeper than a few mm cannot be obtained non-destructively.

When X-rays are irradiated on a sample, not only photoelectrons and fluorescence X-rays but also elastic (Rayleigh) and inelastic (Compton) scattered X-rays are emitted from the sample. The energy of both kinds of scattered X-rays is almost the same as that of incident X-rays, and, therefore, scattered X-rays from deep internal areas can pass through a sample if the energy of incident X-rays is high enough. Many pieces of physical and chemical information on samples are contained in scattered X-rays and can be obtained through quantitative analysis of intensity, energy loss, and scattering angles. To date, both kinds of scattered X-rays are widely used in various fields such as the medical^1–4^ and foods fields^5^ as well as for historical samples^6^ to obtain internal information such as on density and structure. In addition, it was reported that the ratio of the intensities of elastic and inelastic scattered X-rays depends on the effective atomic number (*Z*_*eff*_)^7–9^, and the *Z*_*eff*_ of biomedical samples and oils were determined in an example application^10,11^. Furthermore, sectional observation combined with computed tomography was developed^12,13^, and the *Z*_*eff*_s of biomedical samples were tried using X-rays scattered in the forward direction^14^. Microscopic observation was also performed in combination with confocal micro X-ray scattering, and a sectional image of a polymer layer was obtained^15^. In a practical application, X-ray backscatter imaging used to detect elastic and inelastic back-scattered X-rays from a sample (detected from the front side of a sample) was developed^16^, and it was used for checking the inner structure of infrastructures that X-rays cannot pass through.

To perform *Z*_*eff*_ mapping of deep internal areas from the front side, we developed a novel *Z*_*eff*_ imaging method that uses elastic and inelastic back-scattered X-rays. The ratio of these X-rays is theoretically indicated to depend on the *Z*_*eff*_, and so *Z*_*eff*_ maps can be obtained through a combination of ratio detection and X-ray scanning. The energy of back-scattered X-rays depends on that of incident X-rays, and it can be set as desired. Therefore, *Z*_*eff*_ observation of deep areas can be performed by using high-energy X-rays having high penetration. In addition, cluster analysis of maps of elastic and inelastic back-scattered X-rays also provides a way to perform fine segmentations and to obtain information on the thickness of samples.

To test the feasibility of the novel method, we first measured elastic and inelastic back-scattered X-rays with various kinds of materials at several scattering angles by changing the energy of X-rays use to irradiate the samples [monochromated synchrotron radiation (SR) X-ray]. After optimizing the measurement conditions by using the obtained data, we performed elemental mapping on a phantom and evaluated the accuracy of *Z*_*eff*_ calculated from the ratio of the elastic and inelastic back-scattered X-ray intensities (RC ratio). Finally, we tested the feasibility of using the method for segmentation by analyzing an elastic and inelastic back-scattered X-ray map.

**Figure 1:**
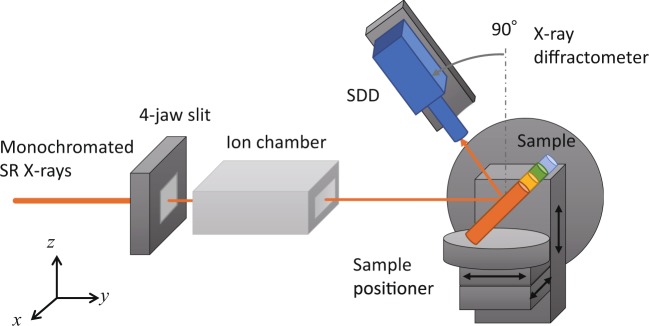
Schematic view of experimental set-up for measuring back-scattered X-rays, which were detected by SDD having high-energy resolution.

**Figure 2:**
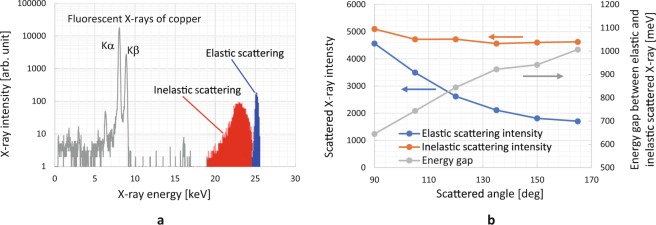
(**a)** Scattered X-ray spectrum of copper rod at scattering angle of 135 degrees using 25-keV SR X-rays. Elastic (blue area) and inelastic (red area) scattered X-rays were clearly separated. RC ratios were calculated by using each area, not peak height. (**b)** Intensities of elastic and inelastic X-rays scattered from rod at different scattering angles using 25-keV SR X-rays.

Back-scattered X-rays were measured by using the experimental set-up shown in Fig. 1. Monochromated SR X-rays were formed into a 1-mm square shape by using an X-ray 4-jaw slit and irradiated onto a sample positioned by using the sample positioner of an X-ray diffractometer. Back-scattered X-rays were detected by a silicon drift detector (SDD). Cylindrical rods (3-mm diameter) made of polyethylene, aluminum, stainless steel (SUS304), and copper were selected as the samples because the wide range of their atomic number was suitable for checking the feasibility of the method. Samples were positioned at an incident angle of 45 degrees. Figure 2a shows an example spectrum for a copper rod obtained at a scattering angle of 135 degrees by using 25-keV SR X-rays. Elastic, inelastic, and fluorescent X-rays were clearly separated owing to the high-energy resolution of the SDD, so a fine RC ratio (blue and red area ratio) could be obtained. The elastic and the inelastic scattering areas were divided at the bottom between both peaks. Figure 2b shows the intensities of the elastic and inelastic X-rays scattered from the rod and the energy gap between the scattered X-rays at different scattering angles using 25-keV SR X-rays. The results show that the intensity for both X-rays depended on the scattering angle and decreased as the angle increased. On the other hand, the energy gap increased as the angle increased expected from Compton effect.

**Figure 3:**
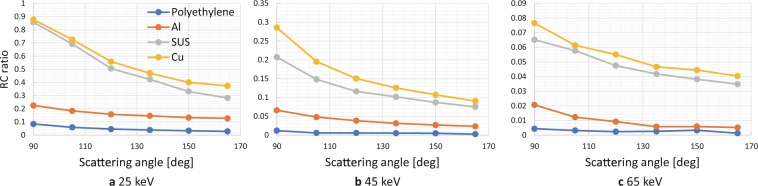
RC ratio of each material (polyethylene, aluminum, SUS, and copper) at different scattering angles (90, 105, …, 165 degrees) using 25-, 45-, and 65-keV SR X-rays. RC ratio of each material depended on element kinds, and their values increased in order of periodic table.

Figure 3 shows the RC ratio of each material at different scattering angles obtained by using 25-, 45-, and 65-keV SR X-rays. The intensity of the scattered X-rays for each material was calculated by using each spectrum area (not the peak value) as shown in Fig. 2a to suppress the error caused by quantum noise. These results show that the RC ratio of each material depended on the kinds of elements, and their values increased in the order of the periodic table, that is, in the order of polyethylene, aluminum, SUS, and copper. In addition, the RC ratio decreased as the scattering angle and X-ray energy increased; however, the order of the RC ratio of each material was kept the same. These results are summarized as follows from the point of view of elemental imaging.

1. The RC ratio depended on the atomic number of the samples, and *Z*_*eff*_ mapping could be performed.
2. The RC ratio decreased with the scattering angle, and a low angle was suitable for fine observation. However, the gaps between the energies of the elastic and inelastic scattering X-rays became smaller as the angle decreased, so the angle has to be optimized in consideration of the energy resolution of the SDD and measurement period.
3. The RC ratio increased as the X-ray energy decreased, and low-energy X-rays were suitable for fine observation. However, the absorption length of the X-rays shortened as the X-ray energy decreased. Therefore, X-ray energy has to be selected in consideration of the depth of the target area.

**Figure 4:**
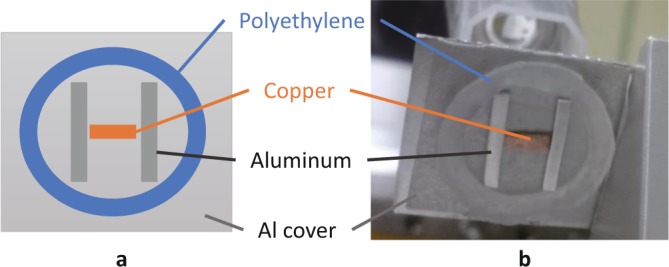
(**a)** Illustration of phantom consisting of polyethylene ring and aluminum and copper blocks covered by aluminum plate with 1-mm thickness. (**b)** Photo of phantom taken from back side.

As a next step, we tested the feasibility of observing a phantom that consisted of a polyethylene ring and aluminum and copper blocks covered by an aluminum plate with a 1-mm thickness, as shown in Fig. 4. A pencil monochromated SR X-ray beam in the shape of a 0.5-mm square was formed by using the X-ray 4-jaw slit and scanned on the phantom by using the sample positioner. The X-rays scattered at each point were detected by the same SDD with a 1-sec counting time at a scattering angle of 135 degrees, and the sample was set at an incident angle of 45 degrees. The scanning step and number of both x- and z-directions were 0.25 mm and 81 points, respectively, and therefore the scanned area was an 20-mm square. The SR X-ray energy was set at 25 keV, which was high enough for transmission through the aluminum cover of the phantom.

**Figure 5:**
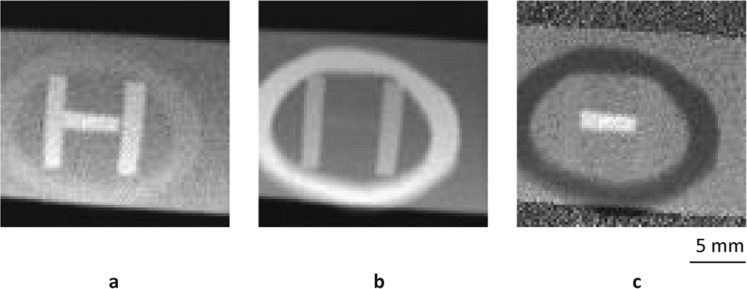
Elastic (**a)**, inelastic (**b)**, and RC ratio (**c)** images. Each material was successfully visualized in order of atomic number in **c** as expected from Fig. 3.

Figure 5 shows the obtained **a** elastic, **b** inelastic, and **c** RC ratio images. The intensity of the scattered X-rays for each material was also calculated by using the corresponding spectrum area as shown in Fig. 2a. Cross-sections of elastic scattering are positively correlated with the atomic number, and therefore, the copper area was visualized with bright contrast in Fig. 5a. On the other hand, cross-sections of inelastic scattering are negatively correlated with the atomic number, and therefore the polyethylene area was visualized with bright contrast in Fig. 5b. The RC ratio was found to be positively correlated with the atomic number as revealed in Fig. 3, and each material was successfully visualized in the order of atomic number, that is, copper, aluminum, and polyethylene, in Fig. 5c as expected. Namely, a *Z*_*eff*_ map of the inner area was successfully obtained by using back-scattered X-rays in front-side observation. Note that the thickness information was canceled out due to the division of the intensity of the scattered X-rays, so the aluminum blocks had the same value as the areas inside and outside the ring that had no blocks in the RC ratio image. In addition, the relative differences in the RC ratio were used as image contrast in Fig. 5c, so the signal from the aluminum plate was simply a background signal and thus disappeared in the area covered by the aluminum plate.

**Figure 6:**
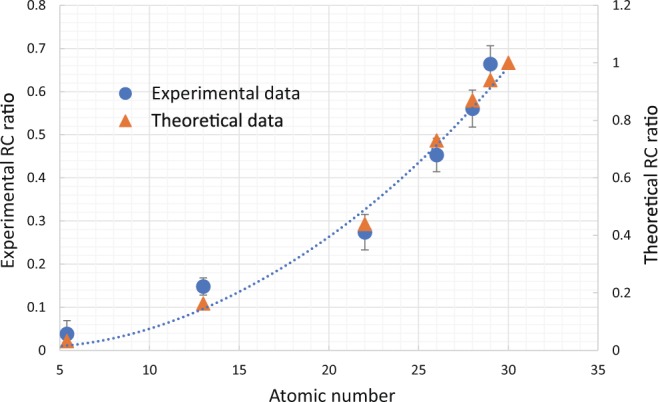
RC ratios of polyethylene, aluminum, titanium, iron, nickel, and copper plates obtained in measurement and theoretical calculation. Ratios for both were almost promotional to square of *Z*_*eff*_, and each material kind could be identified.

A theoretical consideration using a simple model (Thomson-Fermi approximation) showed that the RC ratio was proportional to the square of *Z*_*eff*_^9^. We evaluated the accuracy of *Z*_*eff*_ calculated by using the experimentally measured RC ratio of each material quantitatively. Figure 6 shows the measured and theoretical RC ratios of polyethylene, aluminum, titanium, iron, nickel, and copper plates. The measurement was performed by using the same experimental set-up shown in Fig. 1. The scattering angle was fixed at 135 degrees, and the energy of the SR X-rays was set at 25 keV, and the incident angle of the plates was set at 45 degrees. The theoretical RC ratios were calculated by using the Scattering Angular distribution Plot (SAP) program^17,18^, which is a graphical tool developed at the University of Bologna to compute and plot the angular distribution of scattering kernels and of reflected and transmitted scattering intensities, under the same conditions (135 degrees and 25 keV).

All data points were fit within the error bar except aluminum, and the ratios for both intensities were almost proportional to the square of *Z*_*eff*_. In addition, the variability in the ratio was assumed to be smaller than 1 in high *Z* number region (*Z* > 25), so each kind of material could be identified if the sample was composed of a single element. Note that the scale of each RC ratio differed because the measured RC ratios were calculated using the area ratios in the spectrums as shown in Fig. 2a, while the theoretical ratios were calculated using the peak height ratio of each scattered X-ray. The scale was fit to minimize the least square error between the experimental and theoretical data. The reason for the large error in aluminum was considered to be tiny X-ray diffraction from aluminum.

The approximated quadratic equation used to calculate the ratio between the RC ratio and *Z*_*eff*_ is given as follows by using the least square method.

1 $$RC=0.0009{{Z}_{eff}}^{2}-0.006{Z}_{eff}+0.0189$$

The *Z*_*eff*_ calculated by using the measured ratios of each plate of material, that is, the polyethylene, aluminum, titanium, iron, nickel, and copper plates, were 9.0 (5.4), 15.4 (13), 20.6 (22), 25.5 (26), 28.2 (28), and 30.6 (29), respectively. () show the *Z*_*eff*_ calculated by using the elemental components of the plates. These results show that the average accuracy for *Z*_*eff*_ was estimated to be <±1, except for polyethylene and aluminum, which had an accuracy that was high enough to identify mono-element materials for high *Z* elements (*Z* > 25).

**Figure 7:**
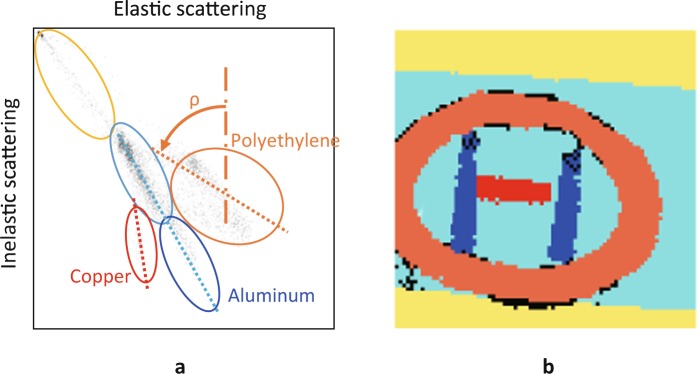
(**a)** Elastic and inelastic X-ray intensity map (EIE map) created by plotting corresponding points of all pixels in images of Fig. 5. (**b)** Image of phantom segmented by using EIE map. Blocks and ring are clearly identified.

Two physical values, elastic and inelastic scattering X-ray intensity, are obtained at the same time at the same pixel (point) with the novel imaging method. Therefore, cluster analysis of a map of both intensities can be applied to perform fine segmentation and obtain additional information in the same way as the segmentation in phase-contrast X-ray imaging^19^. Figure 7a shows an elastic and inelastic X-ray intensity map (EIE map) obtained by plotting the corresponding points of all pixels in the images of Fig. 5. Several groups corresponding to each area (polyethylene, aluminum, and copper) appeared, and segmentation could be performed by clustering all points in the groups manually. Figure 7b shows a segmented image of the phantom. Each of the blocks and ring of material is clearly identified. In addition, by separating the aluminum group into two groups in the middle based on the known information (aluminum composed of plate and blocks), as shown in Fig. 7a, the cover of the phantom corresponding to the cyan group and the aluminum blocks corresponding to the blue group were clearly identified. The intensity of the scattered X-rays for the cover was lower than that for the aluminum blocks because the total thickness of the aluminum of the cover was thinner than that of the blocks. These results show that fine segmentation can be performed, and depth information can be obtained by using an EIE map.

The map also can be used to obtain the *Z*_*eff*_ of each material. The RC ratio of each group can be written as

2 $$RC={\tan }^{-1}(\rho )$$

where *ρ* is the inclination of each ellipse of a group, as shown by the dashed lines in Fig. 7a. The inclinations of polyethylene, aluminum, and copper were estimated to be about 65, 35, and 9 degrees, respectively, and therefore, the ratios were calculated to be 0.04, 0.14, and 0.63, respectively. By using Eq. (1), the *Z*_*eff*_ of each element was obtained to be 9.2, 15.5, and 29.7, which were close to the ideal values.

The newly developed method provides a way to obtain *Z*_*eff*_ information in deep areas in front-side observation, so observing and analyzing the *Z*_*eff*_ s of various samples, such as magnets, electronic devices, and biomedical tissues, are expected to be possible. In addition, fine observation with a μm-order spatial resolution is also expected to be possible with focused X-ray micro beams. Furthermore, methods for non-destructive three-dimensional *Z*_*eff*_ observation that combine tomosynthesis and/or energy scanning methods are waiting to be developed. In addition, more accurate segmentation of materials is expected using machine learning or neural networks on EIE maps.

## Method

When X-rays are irradiated on a sample, elastic (Rayleigh) scattered X-ray, inelastic (Compton) scattered X-ray, fluorescent X-rays, and photoelectrons are generated from the sample. Cross-sections of elastic ${(\frac{d\sigma }{d\Omega })}_{R}$ and inelastic ${(\frac{d\sigma }{d\Omega })}_{C}$ scattering are given as

3 $${(\frac{d\sigma }{d\Omega })}_{R}={|F(q,Z)|}^{2}{(\frac{d\sigma }{d\Omega })}_{Th}$$

4 $${(\frac{d\sigma }{d\Omega })}_{C}=S(q,\,Z){(\frac{d\sigma }{d\Omega })}_{KN}$$

where ${(\frac{d\sigma }{d\Omega })}_{Th}$ and ${(\frac{d\sigma }{d\Omega })}_{KN}$ are Thomson scattering and Klein-Nishina cross-sections, respectively^20^. *F*(*q*, *Z*) is the coherent scatter form factor for element *Z*, *S*(*q*, *Z*) is an incoherent scattering function, and q is a momentum transfer function, given as

5 $$q=\frac{1}{\lambda }\,\sin (\frac{\theta }{2})$$

where *θ* is the scattering angle, and $\lambda$ is the X-ray wavelength. If X-ray energy is lower than 100 keV, Thomson scattering and Klein-Nishina cross-sections can be assumed to have the same value^21^, and therefore the RC ratios of elastic (Rayleigh) and inelastic (Compton) scattered X-rays are finally given as follows.

6 $$RC=\frac{{|f(q,Z)|}^{2}{(\frac{d\sigma }{d\Omega })}_{Th}}{S(q,\,Z){(\frac{d\sigma }{d\Omega })}_{KN}}\propto \frac{{|f(q,Z)|}^{2}}{S(q,\,Z)}$$

Here, *q* can be assumed to be a constant if *λ* and *θ* are fixed, so the RC ratio depends only on *Z*. For composite materials, the effective atomic number (*Z*_*eff*_) can be obtained instead of *Z*. By using a simple model (Thomson-Fermi approximation), it was reported that the RC ratio was proportional to the square of *Z*_*eff*_^7^.

Elastic and inelastic scattering X-ray measurements were carried out by using the X-ray diffractometer shown in Fig. 1 at the beamline BL07 of SAGA Light Source (SAGA-LS) in Tosu and the BL16B2 of SPring-8 in Sayo in Japan. The data shown in Figs. 2a,b, 3a, and 6 were obtained at SAGA-LS, and the remainder of the data were obtained at SPring-8. The white SR X-rays emitted from the SR source were monochromated by using a double-crystal monochrometer [Si(220) at SAGA-LS and Si(111) at SPring-8]. The scattered X-rays were detected by a silicon drift detector (Amptek XR-100SDD) with a 0.5-mil Be window. The energy resolution was about 130 eV, and the number of channels was 2048. The SDD was mounted on the arm of the X-ray diffractometer, and the angle used for detection was adjusted by rotating the arm. The size of the detector area of the SSD was about 25 mm^2^. The two-dimensional observation of Fig. 5 was performed by moving the sample with the sample positioner, which was driven by a stepping motor. The X-rays scattered at each point were detected by using the step scanning method.

## Data Availability

The datasets generated during and/or analyzed during the current study are available from the corresponding author on reasonable request.
